# KtrB-mediated alkaline adaptation drives *Enterococcus faecalis* persistence in the gastrointestinal tract of *Helicoverpa zea*

**DOI:** 10.3389/fmicb.2025.1641331

**Published:** 2025-10-28

**Authors:** Patrick J. Hurd, Vivian Veto, Geneva Bell, Jerreme J. Jackson

**Affiliations:** Department of Biology, University of Northern Iowa, Cedar Falls, IA, United States

**Keywords:** *Helicoverpa zea*, *Enterococcus faecalis*, Host-microbe interactions, alkaline stress, potassium transport, gut microbiome, KtrB, *ntpJ*

## Abstract

*Enterococcus faecalis* is a commensal enteric bacterium capable of surviving in extreme and diverse environments. Here, we characterized the role of the gene *ntpJ*, which encodes the KtrB subunit of the KtrAB Na^+^/K^+^ symporter, during the adaptation of *E. faecalis* to alkaline stress and persistence in *Helicoverpa zea* (corn earworm). We assessed growth kinetics, biofilm formation, surface adhesion, and gastrointestinal persistence *in vivo* using an *E. faecalis* OG1RF mariner transposon mutant (*ntpJ*-Tn). The *ntpJ*-Tn mutant showed delayed entry into mid-log phase growth and biofilm formation under standard and alkaline-adjusted conditions relative to the wild-type strain, while adherence to a low-density substrate was not affected, indicating KtrB-mediated transport was important for early-stage planktonic growth but unnecessary for surface attachment. Interestingly, elevated K^+^ and Na^+^ ions differentially influenced biofilm morphology and the distribution of adherent cells, highlighting an ion-specific response to alkalinity. The *ntpJ*-Tn mutant was undetectable 48 hours following ingestion in the novel non-destructive *H. zea* model, suggesting the loss of KtrB resulted in a persistence defect. These findings reinforce the significance of KtrB-mediated transport in sustaining optimal ionic homeostasis during microbial survival of alkaline stress and demonstrate the efficacy of lepidopteran models for interrogating host-microbe interactions.

## 1 Introduction

*Enterococcus faecalis* is a gram-positive facultative anaerobe and opportunistic pathogen routinely identified among diverse microorganisms in the animal gastrointestinal tract (GIT), ranging from mammals to insects (Martin and Mundt, 1972; Krawczyk et al., 2021). Studies have demonstrated *E. faecalis* to be capable of withstanding a wide range of pH, temperatures, and salinity, highlighting its inherent resilience, and contributing to its persistence in diverse hosts and environments.

The adaptability of *E. faecalis* has made it a useful model for elucidating the mechanisms underlying host-microbe interactions in non-mammalian systems, such as lepidopterans, where the dynamic GIT environment presents unique physiological challenges for transient, resident, and invasive pathogens. Species in the order Lepidoptera, which include caterpillars and moths, have been used extensively to characterize the toxicity of insecticidal proteins produced by *Bacillus thuringiensis* (Bt) (Schnepf et al., 1998; Jurat-Fuentes et al., 2011; Kerns et al., 2023). However, the holometabolous life cycle and proportionately large GIT, genetic tractability, and microbiome plasticity have contributed in recent decades to their emergence as useful models to characterize host-microbe interactions.

The lepidopteran midgut presents a unique anatomical and physiological environmental niche including high luminal alkalinity, elevated potassium concentration, reduced oxygen availability, short retention times, and the presence of a peritrophic matrix that protects the epithelium from mechanical damage, pathogens, and toxins (Dow, 1992; Lehane, 1997; Johnson and Barbehenn, 2000; Brinkmann and Tebbe, 2007). While the broad-range utility of lepidopteran systems for the investigation of host-microbe interactions has increased, studies on the molecular mechanisms underlying persistence in the GIT have not. Moreover, these studies have relied on destructive sampling approaches which prevent longitudinal tracking of persistence dynamics in individuals over time (Tang et al., 2004; Holt et al., 2015; Teh et al., 2016; Mazumdar et al., 2021; Zhang et al., 2022). Given the combined alkalinity and potassium levels in the GIT of the lepidopteran species, it is plausible that proteins involved in osmoregulation and ionic homeostasis contribute to bacterial persistence in this environment.

Initially described in *Enterococcus hirae* strain ATCC 9790, the *ntpJ* gene is located at 3′ end of the vacuolar V_0_V_1_ type Na^+^-ATPase (ntp) operon. This operon comprises 11 open reading frames (ORFs), including *ntpF*, -*I*, -*K*, -*E*, -*C*, -*G*, -*A*, -*B*, -*D*, -*H* and -*J*, and has homologs in the Cyanobacterium *Synechocystis sp*. PCC 6803 as well as many clinical *E. faecalis* isolates. Our BLASTp and tBLASTn queries of the *E. faecalis* OG1RF genome (GenBank: CP002621.1) with accession numbers corresponding to each of the ntp genes in *E. hirae* indicate that 10 of the 11 genes described by Murata et al. are present, with *ntpJ* located ∼1.025 Mbp away from the remaining gene cluster (Murata et al., 1996). These findings suggest *ntpJ* is independently transcribed from the other ntp operon genes and are consistent with a report which showed the variation of potassium transporter architecture across the *Enterococcus* genus was strain specific (Acciarri et al., 2023). The *ntpJ* gene encodes a 49-kDa integral membrane protein with sequence similarity to K^+^-transporters in *Escherichia coli* (TrkH/TrkG) and *Saccharomyces cerevisiae* (Trk1/Trk2), both of which mediate K^+^ uptake (Takase et al., 1994; Schlosser et al., 1995; Murata et al., 1996; Petrezselyova et al., 2011). Northern blot analysis of total RNA extracted from *E. hirae* revealed that elevated intracellular Na^+^ and/or alkaline pH induce polycistronic expression of *ntpJ* along with the other ntp operon genes (Takase et al., 1994; Murata et al., 1996). *NtpJ* encodes KtrB, the translocating subunit of the high-affinity K^+^ uptake systems in *E. hirae* (KtrII) and *E. faecalis* (KtrAB). The *E. faecalis* cytoplasmic nucleotide-sensitive regulator, KtrA, binds to ATP which is followed by a conformational change that allows it to form a KtrAB complex under osmotic stress and alkalinity. Under alkaline conditions, which diminish proton motive force (PMF) and membrane potential (Δψ), the Na^+^-ATPase extrudes Na^+^ to generate a sodium motive force (SMF). This SMF is subsequently utilized by KtrB to drive K^+^/Na^+^ symport (Murata et al., 1996; Kawano et al., 2000, 2001; Berry et al., 2003; Matsuda et al., 2004; Acciarri et al., 2023).

In this report, we characterized the role of KtrB during *E. faecalis* adaptation to alkaline stress and persistence in the *H. zea* GIT. We hypothesize that KtrB facilitates ionic homeostasis, which in turn contributes to survival and persistence in the *H. zea* GIT. To test this hypothesis, we assessed the impact of transposon mutagenesis of *ntpJ* on bacterial growth kinetics in vitro, biofilm formation, surface adhesion, and persistence using a non-destructive frass-based *H. zea* sampling model.

## 2 Materials and methods

### 2.1 Insect sources and rearing conditions

*Helicoverpa zea* eggs, originally sourced from the USDA, were obtained from Benzon Research Inc. (Carlisle, PA). Larvae were reared on a soy-wheat germ diet with vitamins purchased from Frontier Agricultural Sciences (Newark, DE). All diet was autoclaved prior to use in bioassays. Axenic larvae were obtained by sterilizing eggs before hatching to eliminate microbial contaminants following previously described procedures (Jackson et al., 2024). Briefly, eggs attached to a 4 in^2^ substrate piece were submerged for 5 min. in 100 mL of chlorination solution (0.6% Chlorox [vol./vol], 0.1% Triton X-100 [vol./vol.]) contained in a sterile 250 mL polysulfone bottle top filter (ThermoFisher, Waltham, MA) and swirled intermittently. Eggs were rinsed twice with 200 mL autoclaved ddH_2_O and air-dried on Kimwipes inside a Purifier Logic+ Class II Biosafety Cabinet (Labconco, Fort Scott, KS). After drying, eggs were transferred to UV-treated 8 oz. deli cups (Bare, Lancaster, PA) and incubated under standard rearing conditions (25 °C, 16:8 (L:D) photoperiod, 65% RH) inside a Percival Intellus Environmental Controller (Percival, Perry, IA). Following eclosion, neonates were transferred to diet in 1.25 oz. plastic cups (Frontier, Newark, DE) and maintained under standard rearing conditions.

### 2.2 Bacterial strains

*Enterococcus faecalis* OG1RFS wild type and *Enterococcus faecalis ntpJ*-Tn, hereafter referred to as OG1RFS WT and OG1RF *ntpJ-Tn*, respectively, are rifampin- and fusidic acid-resistant derivatives of *E. faecalis* OG1, a clinical isolate of the human oral cavity (Gold et al., 1975; Dunny et al., 1978). OG1RFS WT was isolated by aerobic selection of mid-log phase OG1RF on brain heart infusion agar supplemented with streptomycin, and OG1RF *ntpJ*-Tn was generated by insertion of a mariner transposon encoding chloramphenicol acetyltransferase into the *ntpJ* open reading frame. The mariner transposon-chromosome junction was sequenced using Illumina technology and mapped to the OG1RF reference genome (GenBank CP002621.1) (Dale et al., 2018; Jackson et al., 2024). Kristich et al. (2008) designed the mariner transposon for mutagenesis in *E. faecalis* using a two-plasmid system. The transposable element used to disrupt *ntpJ*, EfaMarTn, consists of green fluorescent protein (*gfp*) and chloramphenicol resistance genes and lacks transcriptional terminators. Inverted terminal repeats flank the two open reading frames and serve as mariner transposase recognition sites. The transposon is carried on the conjugative delivery plasmid (pCJK72), and a separate plasmid (pCKJ55) encoding an inducible transposase is carried by target cells. The absence of transcriptional terminators in EfaMarTn minimizes effects by permitting transcriptional read-through from the *ntpJ* promoter and reducing transcriptional interference of genes immediately downstream (Kristich et al., 2008).

### 2.3 Media preparation

*E. faecalis* OG1RFS wild type and *E. faecalis* OG1RF *ntpJ*-Tn were incubated in a standard yeast-peptone-dextrose (YPD) medium or YPD adjusted to a pH of 10 with 5 M sodium hydroxide (NaOH) or 3.6 M potassium hydroxide (KOH) using a Mettler Toledo FiveEasy Plus pH meter FP20 (Mettler-Toledo, Columbus, OH) and filtered through a 0.22 μm mixed cellulose ester membrane (ThermoFisher, Waltham, MA) in a Purifier Logic+ Class II Biosafety Cabinet. For YPD low-density (0.3% [wt./vol.]) agar plates, the pH was adjusted to 10 with 5 M NaOH or 3.6 M KOH before autoclaving followed by supplementation with glucose (2% [wt./vol.]). When required for selective growth, antibiotics were used at the following concentrations: rifampicin (Rif), 50 μg mL^–1^; streptomycin (Str), 100 μg mL^–1^; and chloramphenicol (Cm), 30 μg mL^–1^). All chemicals were purchased from Fisher Scientific unless otherwise noted. Because Fisher’s product data does not list the element profile, we estimated the total [K^+^] in our alkaline-adjusted YPD on the following dry weight K^+^ content of yeast extract and peptone: Yeast extract, ∼9300 μg K^+^ g^–1^ (ChemicalBook Inc, 2025); Peptone, ∼1.3% K^+^ g^–1^ (Grisp Reseach Solutions, n.d.). Based on a standard YPD formulation including 10 g L^–1^ yeast extract, 20 g L^–1^ peptone, and ∼0.05 mM K^+^ from the titration with 3.6 M KOH, this yields ∼ 353 mg K+ L^–1^ (∼9 mM). Importantly, this does not indicate that which is readily available at the cell surface, nor does it account for K^+^ that may be sequestered/chelated by other media components.

### 2.4 In vitro growth

Planktonic growth of OG1RFS WT and OG1RF *ntpJ*-Tn in standard YPD or YPD adjusted to a pH of 10 with either NaOH or KOH was tested as follows: Strains were incubated overnight in 5 mL YPD containing rifampicin and streptomycin (OG1RFS WT) or rifampicin and chloramphenicol (OG1RF *ntpJ*-Tn) with shaking at 37 °C in a Lab-Line 3527 Benchtop Orbital Incubator Shaker (Lab-Line, Dubuque, IA). Five microliters of each stationary phase culture were added to 995 μL (1:200 dilution) fresh standard or alkaline-adjusted YPD containing antibiotics and 200 μL was pipetted in quadruplicate to a 96-well cell culture-treated flat-bottom microtiter plate (ThermoFisher, Waltham, MA). Growth at 25 °C with continuous medium-level shaking was monitored spectrophotometrically in 15 min. intervals in a BioTek Synergy HT microplate reader (BioTek, Winooski, VT). To account for cultures reaching mid-log phase (O.D._600_ = 0.4) between the 15-minute microplate reader intervals, the equation of the linear region spanning 1 hr. before and after each strain reached an O.D._600_ ≥ 0.4 was used to approximate the total time required post-inoculation to reach mid-log phase. The experiment was repeated in triplicate. The following Equations 1–4 were adapted from (Fernández-Martínez et al., 2024) and used to calculate growth kinetics:

Time from the start of the linear region to reach mid-log phase, where *O*.*D*._*Desired*_ is 0.4, μ is the slope of the linear region, 𝔱 is the elapsed time within the linear region, and *O*.*D*._*Starting*_ is the optical density at the first time point of the linear region.


$$ln\left(O.D._{D\,e\,s\,i\,r\,e\,d}\right)=\left(\mu \right)\times \left(t\right)+ln\left(O.D._{S\,t\,a\,r\,t\,i\,n\,g}\right)$$ (1)

Rearrange the equation to solve for μ, where *O*.*D*._*Starting*_ and *O*.*D*._*Final*_ are the optical densities from the first and last time points of the linear region, respectively, and *h* is the duration of the linear region in hours:


$$\mu =\frac{\left[\ln\left(O.D._{F\,i\,n\,a\,l}\right)\;-\;\ln\left(O.D._{S\,t\,a\,r\,t\,i\,n\,g}\right)\right]}{h}$$ (2)

To solve for the time t required to reach the mid-log phase from the start of the linear region, where and *O*.*D*._*Desired*_ is 0.4:


$$t=\frac{\left[\ln\left(O.D._{D\,e\,s\,i\,r\,e\,d}\right)\;-\;ln\left(O.D._{S\,t\,a\,r\,t\,i\,n\,g}\right)\right]}{\mu }$$ (3)

Total hours (T) to reach mid-log phase, where *Time*_*Starting*_ equals the starting time of the linear region:


$$T=T\,i\,m\,e_{S\,t\,a\,r\,t\,i\,n\,g}+t$$ (4)

### 2.5 Microtiter plate biofilm assays

To test the effects of excess potassium or sodium on static biofilm growth, *E. faecalis* OG1RFS WT and *E. faecalis* OG1RF *ntpJ*-Tn were inoculated into 5 mL of YPD media containing the appropriate antibiotics and incubated overnight with shaking at 37° C. Five microliters of stationary phase cultures were added to 995 μL (1:200 dilution, OD_600_ ≅ 0.005) of the aforementioned standard or alkaline-adjusted YPD media and 200 μL was transferred in quadruplicate to wells of a 96-well flat-bottom microtiter plate. Plates were incubated at 25° C in an Air Jacketed CO_2_ incubator (VWR, Radnor, PA) for 24 or 48 h. To quantify adherent cells, we followed the method previously described with slight modifications (O’Toole, 2011). To stain cells, planktonic and non-adherent cells were removed by inverting the microtiter plates and gently shaking out the media. The plates were then rinsed four times by submerging them in PBS and inverting them to wash away any remaining unattached cells and residual media. After rinsing, the plates were blotted on paper towels and left inverted to air dry at room temperature for 15 min. Adherent cells were stained by adding 125 μL of 0.1% (wt./vol.) crystal violet to each well using a multichannel pipette and incubating for 15 min. at room temperature. The residual stain was removed using the same rinsing procedure described above. Plates were again inverted on fresh paper towels and allowed to air drive overnight. The following day, the crystal violet retained in adherent cell walls was solubilized by adding 125 μL of 30% (vol./vol.) glacial acetic acid to each well and incubating for 15 min. at room temperature. The solubilized dye was transferred to a new microtiter plate, and absorbance at 550 nm was measured using a Synergy HT microplate reader. The data presented represents the average of three independent experiments.

### 2.6 Overlay adhesion assay

*E. faecalis* OG1RFS WT and *E. faecalis* OG1RF *ntpJ*-Tn were inoculated into 5 mL of YPD media containing the appropriate antibiotics and incubated overnight with shaking at 37° C. To test the adhesive properties of the strains, we followed a previously described method with slight modifications (Reynolds et al., 2008). Twenty microliters of stationary phase cultures were pipetted onto the surface of YPD low-density (0.3% [wt./vol.]) alkaline-adjusted agar plates and incubated for 5 days at 25° C in an Air Jacketed CO_2_ incubator (VWR, Radnor, PA). A rectangular piece of commercial plastic wrap (Great Value, Bentonville, AR) was carefully placed over the bacterial colony, allowing it to adhere to the surface by capillary action. Sufficient excess plastic was left on both sides to enable removal without disturbing the agar. After 10 s, the plastic was gently and evenly lifted from both sides, inverted, and placed on an 8” × 11.5” laminated piece of black construction paper for imaging using a Canon EOS Rebel Tx Digital SLR Camera (Canon, Tokyo, JP) to capture the distribution of non-adherent cells. Images of the cells remaining on the agar surface were captured to document the adherent population.

### 2.7 Persistence bioassays

The association of OG1RFS WT and OG1RF *ntpJ*-Tn with *H. zea* larvae was performed as previously described (Jackson et al., 2024). Briefly, on day seven post-eclosion, larvae were fasted overnight, and OG1RFS WT and OG1RF *ntpJ*-Tn were each inoculated into 5 mL standard YPD with antibiotics and grown overnight as described previously. The next day, 1 mL of each stationary phase culture was pelleted by centrifugation (17,000 × *g* for 5 min.) at room temperature in an AccuSpin Micro 17R Microcentrifuge (ThermoFisher, Waltham, MA) then washed once in sterile phosphate-buffered saline (PBS, 137 mM NaCl, 2.7 mM KCl, 10 mM Na_2_HPO4, 1.8 mM KH_2_PO_4_) pH 7.4. Pellets were resuspended in 1 mL PBS (Tube 1: ∼10^9^ CFU mL^–1^), followed by the transfer of 100 μL to a new tube containing 900 microliters of PBS (Tube 2: ∼10^8^ CFU mL^–1^). One hundred microliters of each strain were added to 900 microliters of PBS (∼10^7^ CFU mL^–1^). One hundred microliters of each strain were pipetted onto the surface of general-purpose lepidopteran diet, which had been amended with 50 μg mL^–1^ rifampicin and previously dispensed into 128-well bioassay trays (Frontier Agricultural Sciences, Newark, DE). After gently swirling to evenly coat the diet surface with bacteria, the bioassay trays were dried for ∼4 h. in a Purifier Logic+ Class II Biosafety Cabinet. After drying, three fasted larvae were transferred to diet surface contaminated with each strain (one larva per well), after which the trays were covered with ventilated adhesive seals (Frontier, Newark, DE) and incubated in a growth chamber under the previously described standard rearing conditions. The density of each strain applied to the diet was estimated by plating on selective media and recorded (CFU mL^–1^). Larvae were transferred to fresh diet amended with 50 μg mL^–1^ rifampicin in 1.25 oz. plastic cups (Frontier, Newark, DE) at twenty-four and forty-eight-hours post-association using sterile forceps and without disturbing frass (feces) and returned to the growth chamber. Frass from each larva remaining in the bioassay tray and the 1.25 oz. plastic cups was transferred using sterile blunt-end forceps into an autoclaved low-retention microcentrifuge tube (ThermoFisher, Waltham, MA), and weighed using an OHAUS Analytical Balance (OHAUS, Parsippany, NJ). Each frass sample was diluted in sterile PBS (1:10 [wt./vol.]) and vortexed for 10 s every 15 min. for one hour to liberate bacteria. The top soluble portion of each frass homogenate was then serially diluted in sterile PBS (1:10 [vol./vol.]), and 100 μL was spread using autoclaved borosilicate glass beads (ThermoFisher, Waltham, MA) on YPD agar plates containing rifampicin and streptomycin for OG1RFS WT or rifampicin and chloramphenicol for OG1RF *ntpJ*-Tn. Plates were incubated at 37 °C for 24–48 h before CFUs were counted. The log-transformed viable counts of each strain were reported as the average log_10_ CFU per gram of frass. The experiment was repeated in triplicate.

### 2.8 Statistical analysis

Statistically significant differences were analyzed using the Student’s *t*-test (overall significance level = 0.05). The comparison between the growth of OG1RFS WT in KOH and NaOH failed the Shapiro-Wilk normality test, so we determined a significant difference using the Mann-Whitney Rank Sum test (overall significance level = 0.05). The comparisons between OG1RFS WT and OG1RF *ntpJ*-Tn biofilm formation in YPD-10 (NaOH) after 24 h, in YPD after 48 h, and the Day 2 comparison during the persistence bioassay failed the Brown-Forsythe equal variance test, so we determined significant differences using Welch’s *t*-test (overall significance = 0.05). When biofilm data failed the Shapiro-Wilk normality test, we determined statistically significant differences using Mann-Whitney Rank Sum tests (overall significance = 0.05). Data analysis and graph generation were performed using SigmaPlot v.14.0 (Systat, Palo Alto, CA). Effect sizes were calculated in Microsoft Excel as Cohen’s *d*, defined as the absolute mean difference divided by the pooled standard deviation (Cohen, 1988).

## 3 Results

### 3.1 Disruption of *ntpJ* increases sensitivity to alkaline stress during planktonic growth

**FIGURE 1:**
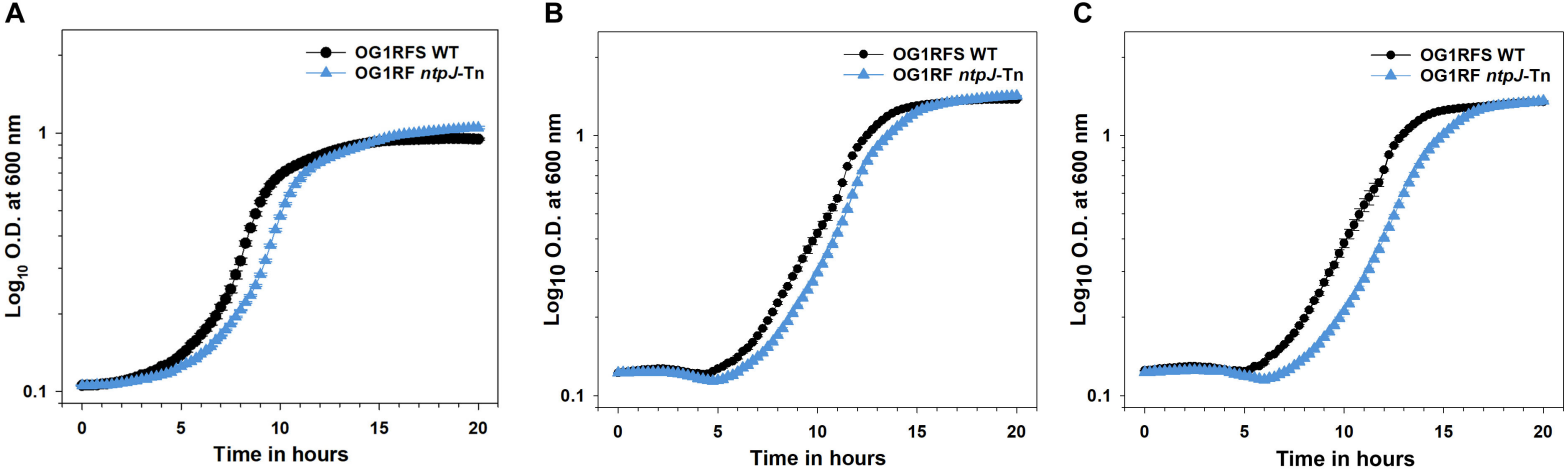
Planktonic growth of *E. faecalis* strains in standard and alkaline-adjusted YPD at 25°C. **(A)** Growth of *E. faecalis* strains in standard YPD; **(B)** Growth of *E. faecalis* strains in YPD adjusted to pH 10 with KOH; **(C)** Growth of *E. faecalis* strains in YPD adjusted to a pH 10 with NaOH. Error bars represent the standard error of the means of O.D._600_ at each time point.

**FIGURE 2:**
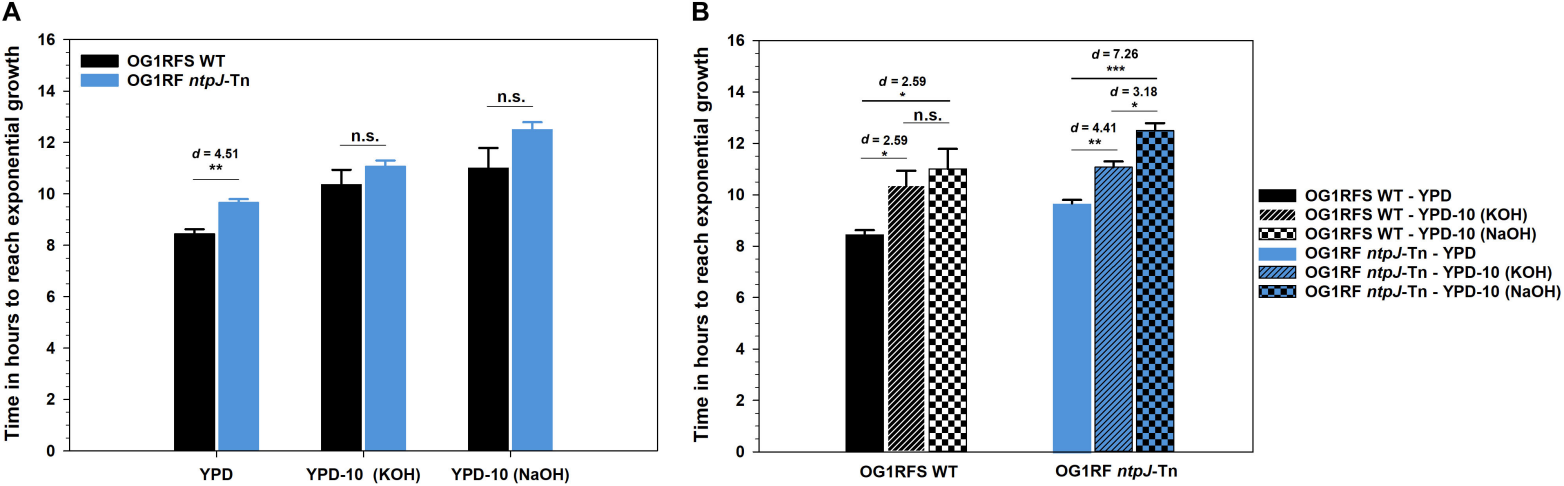
Elapsed time for *E. faecalis* strains to achieve mid-log phase growth in standard and alkaline-adjusted YPD at 25 °C. **(A)** Comparison of *E. faecalis* OG1RFS WT relative to *E. faecalis* OG1RF *ntpJ*-Tn; **(B)** The effects of alkaline-adjusted media on *E. faecalis* strains. Error bars represent the standard error of the means (*p < 0.05, **p < 0.01, ***p < 0.001; Student’s *t*-test, *d* = effect size, n.s. = not significant).

To assess the role of *ntpJ* expression in the alkaline stress response, *E. faecalis* OG1RFS WT and *E. faecalis* OG1RF *ntpJ*-Tn were cultured in standard YPD and YPD adjusted to pH 10 using either KOH or NaOH. Final cell densities (O.D._600_) did not differ between the wild-type and *ntpJ*-Tn mutant strains under any condition indicating the *ntpJ* disruption does not affect the overall growth yield (Figure 1). However, a statistically significant difference in the time required to reach the mid-log phase was observed between the strains in standard YPD medium (*p* = 0.005, *d* = 4.51) indicating the wild-type strain grew much faster than the *ntpJ*-Tn mutant. No significant differences in this parameter were detected between the strains when cultured in YPD adjusted to pH 10 with either KOH or NaOH (Figure 2A). Relative to its growth in standard YPD, the wild-type strain showed significant delays in reaching the mid-log phase in YPD adjusted to 10 with KOH (*p* = 0.034, *d* = 2.59) and NaOH (*p* = 0.033, *d* = 2.59). Similarly, the *ntpJ*-Tn mutant showed significant delays in both KOH (*p* = 0.006, *d* = 4.41) and NaOH (*p* = 0.018, *d* = 7.26) conditions, indicating a strong sensitivity to alkaline stress (Figure 2B). These results suggest that alkaline conditions decrease the growth advantage of *ntpJ* expression by the wild-type strain. While the wild-type strain did show delays under alkaline stress, these delays were more pronounced in the *ntpJ*-Tn mutant. This indicates that KtrB contributes to the ability of *E. faecalis* to adapt to elevated pH and that its disruption results in heightened sensitivity to alkaline conditions during exponential growth.

### 3.2 Alkaline stress moderates the biofilm defect of the *E. faecalis ntpJ* mutant

**FIGURE 3:**
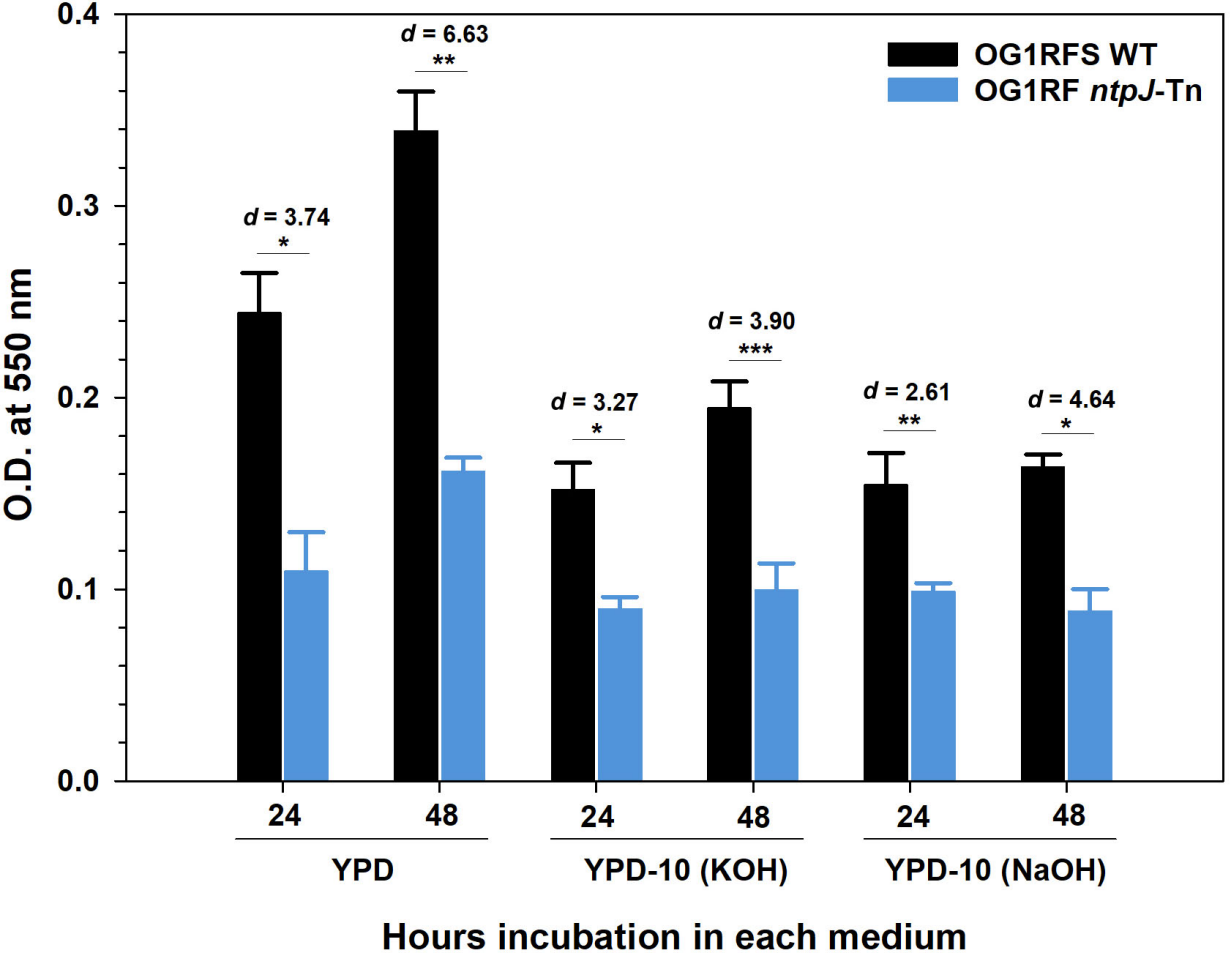
Biofilm formation by *E. faecalis* in standard and alkaline-adjusted YPD for 24 h and 48 h at 25 °C. Error bars represent the standard error of the means (*p < 0.001, Student’s *t*-test; **p < 0.001, Welch’s t-test; ***p < 0.001, Mann-Whitney Rank Sum test; *d* = effect size, n.s. = not significant).

Early-stage biofilm formation by *E. faecalis* OG1RFS WT and *E. faecalis* OG1RF *ntpJ*-Tn was assessed in standard YPD and YPD adjusted to pH 10 using either KOH or NaOH using the crystal violet microtiter plate assay. In standard YPD, the wild-type strain formed significantly more biofilm than the mutant at both 24 h (*p* = 1.36e^–6^, *d* = 3.74) and 48 h (*p* = 5.09e^–7^, *d* = 6.63), indicating a large effect size. The differences persisted in alkaline conditions, but they were less pronounced. In KOH-adjusted YPD, significant differences were observed at 24 h (*p = 1.28e^–5^, *d* = 3.27) and 48 h (***p < .001, *d* = 3.90), while in NaOH-adjusted YPD, differences remained significant at 24 h (**p = 8.22e^–4^, *d* = 2.61) and 48 h (*p = 3.41e^–6^, *d* = 4.64). These results suggest that disruption of *ntpJ*, and the loss of KtrB, significantly impairs biofilm formation, the magnitude of which is reduced under alkaline stress (Figure 3).

### 3.3 *E. faecalis* exhibits increased adherence to agar under alkaline conditions

**FIGURE 4:**
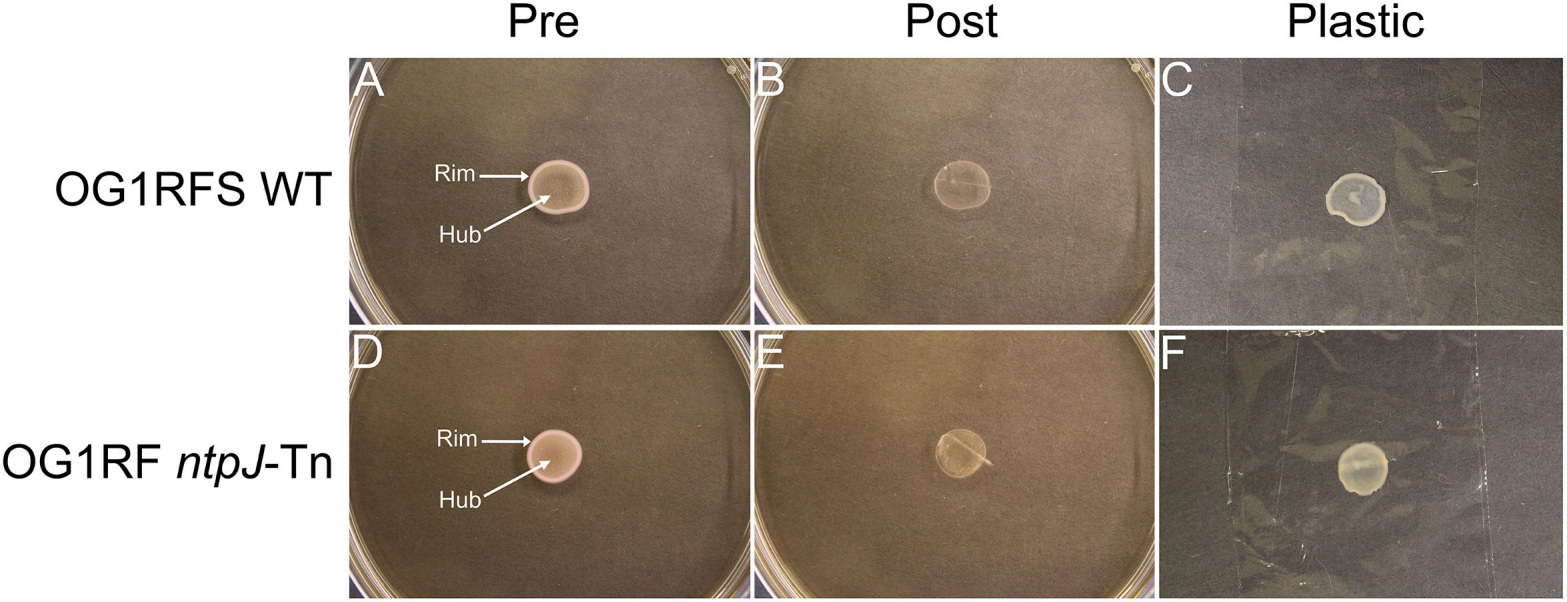
Overlay adhesion assay of *E. faecalis* strains on standard YPD low-density agar. **(A,D)** *E. faecalis* cells before overlay with plastic wrap; **(B,E)** *E. faecalis* cells remaining adhered to agar following removal of plastic wrap; **(C,F)** *E. faecalis* cells removed by plastic wrap.

**FIGURE 5:**
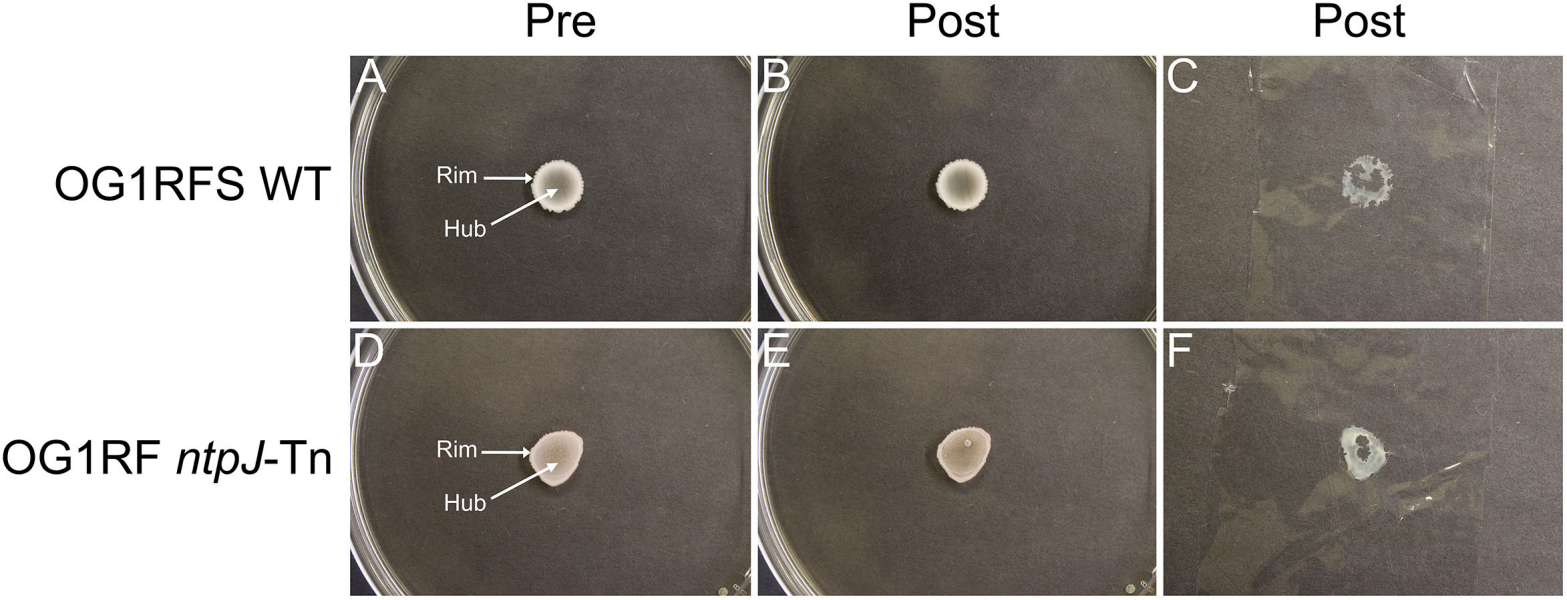
Overlay adhesion assay of *E. faecalis* strains on YPD low-density agar adjusted to a pH of 10 with KOH. **(A,D)** *E. faecalis* cells before overlay with plastic wrap; **(B,E)** *E. faecalis* cells remaining adhered to agar following removal of plastic wrap; **(C,F)** *E. faecalis* cells removed by plastic wrap.

**FIGURE 6:**
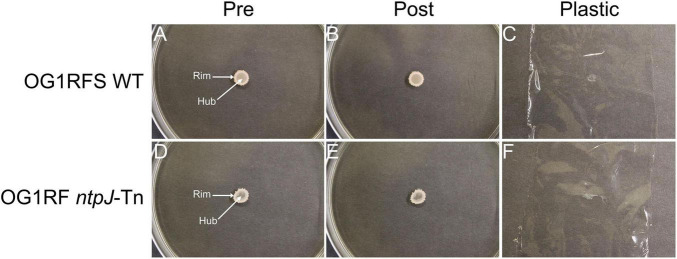
Overlay adhesion assay of *E. faecalis* strains on low-density agar adjusted to a pH of 10 with NaOH. **(A,D)** *E. faecalis* cells before overlay with plastic wrap; **(B,E)** *E. faecalis* cells remaining adhered to agar following removal of plastic wrap; **(C,F)** *E. faecalis* cells removed by plastic wrap.

To investigate the influence of growth substrate and pH on biofilm formation and surface adhesion, an overlay adhesion assay was performed with *E. faecalis* OG1RFS WT and *E. faecalis* OG1RF *ntpJ*-Tn biofilms grown on three types of YPD low-density agar: standard (unaltered pH ∼6.8), pH 10 adjusted with KOH, and pH 10 adjusted with NaOH. Both strains formed biofilms of similar circumference with visually more dense regions around the biofilm rim on standard YPD (Figures 4A, D). Following the overlay procedure, both strains showed reduced adherence to the agar surface. The plastic lifted a substantial amount of nonadherent cell biomass in the rim and hub regions, suggesting weak adherence (Figures 4B, C, E, F). When grown on YPD agar adjusted to pH 10 with KOH, both strains formed biofilms with visually enhanced rim cell density compared to biofilms formed on standard YPD agar (Figures 5A, D). Post-overlay, cells in the rim and hub regions of both strains displayed enhanced adhesion to the agar compared to post-overlay cells under standard conditions (Figures 5B, E). A large majority of nonadherent cells lifted with the plastic were from the rim region of both biofilms (Figures 5C, F). In contrast, biofilms grown on YPD low-density adjusted to pH 10 with NaOH, both strains formed biofilms with smaller circumferences compared to those formed on either standard or KOH-adjusted agar (Figures 6A, D). While these biofilms were the smallest in circumference, they showed the strongest adhesion post-overlay, with most of the cells in the rim and hub regions staying attached to the agar (Figures 6B, C, E, F). These results suggest that adhesion was dependent not only on pH but also on the chemical nature of the base used to adjust pH, as NaOH-adjusted conditions promoted the strongest adherence in both strains in a *ntpJ*-independent manner.

### 3.4 Disruption of *ntpJ* impairs persistence in the *H. zea* GIT

**FIGURE 7:**
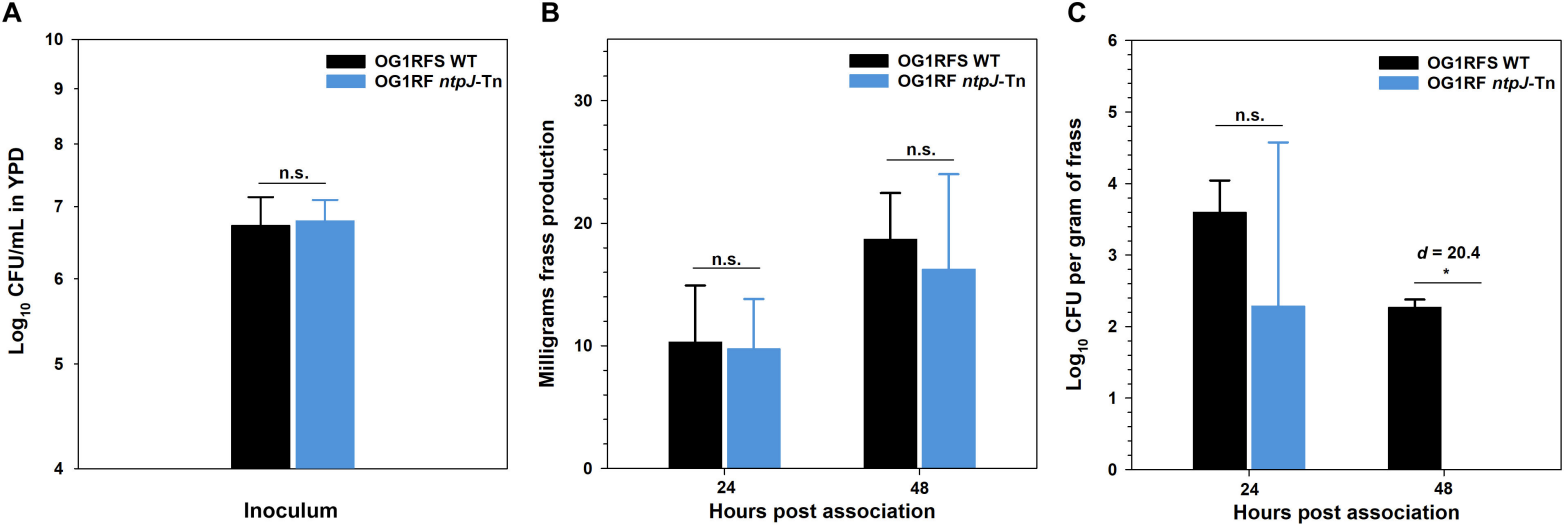
KtrB transport is required for *E. faecalis* persistence in the *H. zea*. **(A)** Inoculum density of *E. faecalis* strains applied to larval diet before ingestion by *H. zea* larvae; **(B)** Frass production by *H. zea* larvae 24 h and 48 h post ingestion of *E. faecalis* strains; **(C)** Density *E. faecalis* strains in the *H. zea* GIT 24 h and 48 h post-ingestion. Error bars represent the standard error of the means (*p < 0.05, Welch’s *t*-test, *d* = effect size, n.s. = not significant).

To assess the role of *ntpJ* expression during *E. faecalis* persistence within the alkaline GIT of *H. zea* larvae, both strains were applied to the surface of the larval diet and non-invasively ingested. At 24 h post-feeding, there were no significant differences between stains in terms of inoculum density, frass production, or bacterial density (Figures 7A–C). By 48 h post-feeding, there was an increase in frass production by larvae fed each strain but no significant differences between groups (Figures 7B–C). However, *E. faecalis* OG1RF *ntpJ*-Tn was undetectable at this time point (*p = 0.031, *d* = 20.4), which suggests the loss of KtrB function reduces tolerance to the alkaline environment of the lepidopteran GIT.

## 4 Discussion

In this study, we demonstrate that KtrB–the integral membrane component of the two-component KtrAB Na^+^/K^+^ symporter encoded by *ntpJ*–contributes to *E. faecalis* adaptation and persistence in the alkaline environment of lepidopteran GIT. The data suggest that KtrB is essential for the planktonic progression into mid-log phase growth and biofilm formation in vitro but is not required for adhesion to semi-solid substrates. The *ntpJ* transposon mutant (OG1RF *ntpJ*-Tn), which lacks a functional KtrB, exhibits a persistence defect in the *H. zea* GIT after 48 h. We hypothesize that KtrB facilitates *E. faecalis* growth under alkaline conditions by maintaining osmotic balance during the initial stages post-ingestion.

The *E. faecalis* OG1RF genome encodes four primary K^+^ transport systems, including two regulator of conductance of K^+^ (RCK)-gated channels (KtrAB and KtrAD) and two K^+^/H^+^ symporters (Kup and KimA). A comparative analysis of the *E. faecalis* OG1RF genome (454 pyrosequencing, Solexa, Sanger) by Bourgogne et al., 2008, BLAST (BLASTp, and tBLASTn) against *E. faecalis* V583 *kdpA-E* protein sequences, and KEGG/Orthology analyses confirmed the absence of the *kdpFABC* operon and its regulatory system, *kdpDE* (Bourgogne et al., 2008).

To test the functional role of KtrB (*ntpJ*) during growth in vitro under neutral conditions, OG1RFS wild type and OG1RF *ntpJ*-Tn were inoculated into standard YPD (pH 6.8-7) and monitored spectrophotometrically. As shown in Figure 1A and Figure 2A, OG1RF *ntpJ*-Tn exhibited a significant delay in the progression to mid-log phase relative to the wild-type strain, suggesting the combined K^+^ influx through KtrD, Kup, and KimA was insufficient to compensate for the loss of KtrB and restore wild-type growth kinetics and supporting a role for KtrB as the primary K^+^ transporter under K^+^-limiting conditions. These results are consistent with Acciarri et al., 2023 (Figure 2) in which an *E. faecalis* JH2-2 derivative (Δ*kup*Δ*ktrA*) experienced a significant growth defect relative to the wild type in mLBG at neutral pH, and align with previous reports detailing that KtrB provides the highest-affinity for K^+^ influx under neutral conditions, followed by Kup and KimA which provide progressively lower-affinity backup K^+^ influx (Kawano et al., 2000; Petrezselyova et al., 2011; Gries et al., 2013; Acciarri et al., 2023).

Under alkaline conditions, *Enterococcus hirae* and *E. faecalis* compensate for the loss of proton motive force (PMF) by activating the V_0_V_1_ type Na^+^-ATPase (V-ATPase) which utilizes the energy from ATP hydrolysis to pump Na^+^ to the extracellular environment. The Na^+^ gradient (sodium motive force, SMF) drives K^+^ influx via KtrB restoring pH and osmotic balance (Kobayashi et al., 1986; Ikegami et al., 1999; Krulwich et al., 2011). To test the functional role of KtrB under alkaline-adjusted conditions, OG1RFS wild type and OG1RF *ntpJ*-Tn were inoculated into YPD adjusted to a pH of 10 with either 3.6 M KOH or 5 M NaOH and monitored spectrophotometrically. We hypothesized that collapse of PMF would impair H^+^-coupled symport of K^+^ through Kup and KimA. Simultaneously, elevation of the extracellular [K^+^] from KOH dissociation would place the burden of K^+^ influx on KtrD, potentially causing OG1RF *ntpJ*-Tn growth defects. As shown in Figure 1B, OG1RF *ntpJ*-Tn did not exhibit a growth delay in the progression to mid-log phase relative to the wild-type strain, suggesting the elevated extracellular [K^+^] from KOH dissociation allowed KtrD and, potentially, Kup and KimA to compensate for the loss of KtrB and restore wild-type growth kinetics despite the collapse of PMF. These results are consistent with findings of Acciarri et al., 2023 (Figure 2C), which demonstrated K^+^ supplementation of mLBG at pH 9 partially restored the growth defects of KtrB- and KtrD-deficient *E. faecalis* JH2-2 derivatives. Combined, these data substantiate the compensatory roles of alternative K^+^ transporters in the absence of SMF-driven KtrB-mediated K^+^ influx. We extended our analysis of the role of KtrB under alkaline conditions to include growth in YPD adjusted to a pH of 10 using NaOH. Under these conditions, the K^+^ concentration remained at baseline (see Figure 1A), while we propose the addition of NaOH collapsed the PMF and simultaneously elevated the extracellular [Na^+^]. Interestingly, Figure 1C shows that OG1RF *ntpJ*-Tn exhibited no significant growth defect relative to the wild-type, suggesting the hyperpolarized membrane potential (ΔΨ) driven by the V-ATPase in tandem with Na^+^ from NaOH dissociation was sufficient to create an electrical gradient that could drive K^+^ influx through KtrD, Kup and KimA under K^+^-limited conditions. Consistent with our observation and subsequent proposal that hyperpolarized ΔΨ contributed to the growth of OG1RF *ntpJ*-Tn in NaOH-adjusted YPD, Acciarri et al., 2025 (Figure 5) demonstrated that *E. faecalis* JH2-2 double mutants (Δ*ktr*AΔ*kimA* → Kup^+^ and Δ*ktr*AΔ*kup* → KimA^+^) can sustain growth in a modified sodium Spizizen medium at near neutral pH (7.0–8.0) in which the pH difference across the plasma membrane is minimized and ΔΨ serves as the primary driver of Kup- and KimA-mediated K^+^/H^+^ symport. In alkaline-adjusted YPD, both *E. faecalis* strains showed similar progression to the mid-log phase, despite exhibiting differential sensitivity to ionic stress evidenced by the effect sizes. The wild-type strain was moderately sensitive to both ions, whereas the *ntpJ*-Tn mutant was more sensitive to Na^+^ than K^+^ (Figure 2). While these data revealed a non-significant delay in the progression to the mid-log phase by the *ntpJ*-Tn mutant in alkaline-adjusted media supplemented with K^+^ or Na^+^, the competition with other enteric microorganisms for nutrients and the peristaltic activity of the host GIT would potentially amplify this phenotype. Moreover, the elevated differential sensitivity of the *ntpJ*-Tn mutant suggests that, in addition to Na^+^-dependent K^+^ uptake, KtrB may play a broader role in ionic stress adaptation. The lack of transcriptional terminators in EfaMarTn would allow the native *ntpJ* promoter to drive read-through into the *napA* open reading frame, reducing the likelihood of a polar effect. Furthermore, studies on the *E. faecalis* strain ATCC 33186 transcriptional response to alkaline conditions (pH 10) showed that *napA* expression is downregulated ∼1.64-fold compared to controls (pH 7.4) (Ran et al., 2015). In combination, the transcriptional read-through and alkaline-induced repression of *napA* suggest NapA did not contribute to these phenotypes.

The *ntpJ*-Tn mutant showed significantly impaired early-stage biofilm formation relative to the wild-type strain under all conditions tested. In standard YPD, the density of biofilms formed by the wild-type strain was significantly larger than those formed by the *ntpJ*-Tn mutant at 24 h and 48 h, supported by increasing effect sizes. This biofilm defect was consistent but diminished under the alkaline-adjusted conditions (Figure 3). Physiologically, attempts to maintain K^+^ and Na^+^ homeostasis may underlie this observation. As mentioned earlier, the compensatory pumping of Na^+^ by the V_0_V_1_ type Na^+^-ATPase to establish SMF under alkaline conditions would reduce the availability of ATP for cell replication–a phenotype that would be exacerbated in the *ntpJ*-Tn mutant leading to reduced biofilm density (Kobayashi et al., 1986; Ikegami et al., 1999). This agrees with the results of the planktonic growth experiments in which the *ntpJ*-Tn mutant showed a growth delay in standard YPD relative to the wild-type strain and suggests that an indirect consequence of reduced planktonic fitness is impaired early-stage biofilm formation. Several previous studies including those in *Mycobacterium tuberculosis*, *Staphylococcus aureus*, and *Pseudomonas aeruginosa* have adopted mariner transposon-based approaches for genome-wide mutagenesis and the subsequent identification of genes driving phenotypes of interest (Sassetti et al., 2001; Gallagher et al., 2011; Fey et al., 2013). We tested the effects of high pH (pH 10) in combination with elevated [K^+^] or [Na^+^] on the growth dynamics of an *E. faecalis ntpJ*-Tn, allowing us to isolate the phenotypic consequences of KtrB disruption under pH and ionic stress.

It was demonstrated in a previous study that *Saccharomyces cerevisiae* biofilm mats exhibit spatially distinct regions, a non-adherent rim, and an adherent hub, influenced by adhesin expression, glucose concentration, and pH gradients. Specifically, Flo11p was shown to be essential for both biofilm formation and adhesion, while glucose concentration and pH were higher in the rim relative to the hub (Reynolds et al., 2008). We adapted the yeast overlay adhesion assay described in the study to further compare the *E. faecalis* OG1RFS WT and OG1RF *ntpJ*-Tn biofilms grown on standard and alkaline-adjusted low-density agar. Our results indicate that environmental pH and the chemical properties of the base used to adjust pH influence biofilm morphology and surface adhesion. Both strains formed weakly adherent biofilms on standard YPD supporting a uniform distribution of metabolically active cells in the rim and hub regions (Figure 4). In contrast, biofilms grown on KOH-adjusted YPD agar showed stronger adherence in the hub relative to the rim, suggesting that nutrient depletion in the hub promoted stronger surface attachment. Most notably, both strains showed the strongest uniform adhesion on NaOH-adjusted YPD agar, suggesting that Na^+^ ions may alter nutrient utilization in a manner that enhances attachment or promotes stronger surface attachment directly, the latter of which is consistent with a previous report demonstrating that environmental factors, such as bile salts and ionic composition, influence *E. faecalis* surface charge heterogeneity enhancing adhesion independently of known adhesive proteins (van Merode et al., 2006) (Figure 6).

In studies of continuous flow cultures, Rolf Freter demonstrated that early-stage adaptation to environmental stress and efficient acquisition of limiting nutrients are critical for establishing stable populations in the mucosal layer of the mammalian GIT (Freter, 1981, 1988; Freter et al., 1983). Relative to mammalian model systems, the GIT of lepidopteran larvae presents a stringently alkaline environment that poses significant physiological challenges to transient and stably colonized microorganisms. The alkalinity of the *H. zea* midgut, with high luminal pH and K^+^, is known to disrupt proton motive force and challenge bacterial ionic homeostasis (Kakinuma, 1987; Dow and Harvey, 1988; Dow, 1992; Fallingborg, 1999). In the *H. zea* bioassays, we demonstrate that KtrB promotes *E. faecalis* persistence in the alkaline GIT of *H. zea*. While frass production by all larvae did not differ and the wild-type and mutant strains were initially recovered at similar levels 24 h post-ingestion, the *ntpJ*-Tn mutant was undetectable by 48 h, indicating a significant persistence defect. Dow and Harvey (1988) used flame photometry to estimate the total and active [K+] in the *Manduca sexta* (tobacco hornworm) midgut to be 204 ± 6 mM and 83 ± 1, respectively (Dow and Harvey, 1988). To date, there have been no direct measurements of the [K^+^] concentration in the *H. zea* GIT. However, given the similarity in the functional mechanisms used by goblet cells to maintain alkaline pH in the lepidopteran midgut, it is appropriate to use as a proxy the experimentally determined concentrations in *M. sexta*.

While the in vitro experiments suggest that the elevated [K^+^] in the GIT would allow the *E. faecalis* OG1RF *ntpJ*-Tn mutant to compensate for PMF collapse, the rapid turnover of intestinal contents further exploits the loss of KtrB and explains the observed persistence defect. Furthermore, we would expect the elevated [K^+^] in the *H. zea* midgut lumen to fully saturate all four K^+^ transporters (e.g., KtrD, KtrB, Kup, and KimA). Because the midgut is also extremely alkaline, the collapsed PMF would significantly diminish the symporter activities of Kup and KimA even with an abundant supply of K^+^. Under the same conditions, the transport activities of KtrB and KtrD would depend on whether the epithelial cells are able to maintain a negative potential in the cytoplasm, which would draw in K^+^. The collapsed PMF and subsequent reduced Kup- and KimA-mediated H^+^/K^+^ symport, the downregulated expression of kimA, and the decreased electrogenic import of H^+^ and K^+^ by KtrD force *E. faecalis* to rely primarily on KtrB under alkaline conditions (Acciarri et al., 2025). The loss of KtrB, consequently, leads to significant persistence defects in the *H. zea* GIT. These findings support the hypothesis that the KtrB, the integral membrane component of the KtrAB potassium uptake system, plays a significant role in maintaining osmotic balance and pH under these conditions. That the *ntpJ*-Tn mutant did not persist beyond 24 h suggests that K^+^ translocation by KtrB is essential for maintaining optimal ionic balance. Furthermore, while both strains showed increased surface attachment to low-density agar, the persistence bioassay results suggest that amplification of the planktonic growth differences in vivo limits the density of the planktonic population that may enter adherent biofilms (Figures 6, 7). This observation also agrees with previous work using this non-destructive model, suggesting that multiple genetic signaling pathways mediate survival and persistence in lepidopteran GIT (Jackson et al., 2024).

In conclusion, these data demonstrate that KtrB-mediated K^+^ and Na^+^ transport plays a significant role in the adaptation of *E. faecalis* to alkaline stress and its ability to withstand the dynamic and inherently alkaline *H. zea* GIT. Loss of KtrB impaired early-stage planktonic growth, biofilm formation in vitro, and persistence *in vivo*. While transport by KtrB was not essential to the surface adhesion of biofilms on low-density agar, its role during ionic homeostasis supported *E. faecalis* persistence in the *H. zea* GIT. These results emphasize the importance of K^+^ and Na^+^ transport systems during bacterial persistence in alkaline environments and highlight the efficacy of the non-destructive model for functionally assessing bacterial circuitry underscoring GIT persistence.

## Data Availability

The original contributions presented in this study are included in this article/supplementary material, further inquiries can be directed to the corresponding author.
